# The Role of Genetically Modified Human Feeder Cells in Maintaining the Integrity of Primary Cultured Human Deciduous Dental Pulp Cells

**DOI:** 10.3390/jcm11206087

**Published:** 2022-10-15

**Authors:** Natsumi Ibano, Emi Inada, Shinji Otake, Yuki Kiyokawa, Kensuke Sakata, Masahiro Sato, Naoko Kubota, Hirofumi Noguchi, Yoko Iwase, Tomoya Murakami, Tadashi Sawami, Yoshito Kakihara, Takeyasu Maeda, Miho Terunuma, Yutaka Terao, Issei Saitoh

**Affiliations:** 1 Department of Pediatric Dentistry, Asahi University School of Dentistry, Mizuho-shi 501-0296, Japan; 2 Department of Pediatric Dentistry, Graduate School of Medical and Dental Sciences, Kagoshima University, Kagoshima 890-8544, Japan; 3 Division of Pediatric Dentistry, Department of Oral Health and Development Sciences, Tohoku University Graduate School of Dentistry, Sendai 980-8575, Japan; 4 Division of Pediatric Dentistry, Faculty of Dentistry, Graduate School of Medical and Dental Sciences, Niigata University, Niigata 951-8514, Japan; 5 Department of Genome Medicine, National Center for Child Health and Development, Tokyo 157-8535, Japan; 6 Section of Gene Expression Regulation, Frontier Science Research Center, Kagoshima University, Kagoshima 890-8544, Japan; 7 Department of Regenerative Medicine, Graduate School of Medicine, University of the Ryukyus, Nishihara 903-0215, Japan; 8 Department of Dentistry for the Disabled, Asahi University School of Dentistry, Mizuho-shi 501-0296, Japan; 9 Kyoto Dental Service Center Central Clinic, Kyoto 604-8418, Japan; 10 Division of Dental Pharmacology, Graduate School of Medical and Dental Sciences, Niigata University, Niigata 951-8514, Japan; 11 Center for Advanced Oral Science, Graduate School of Medical and Dental Sciences, Niigata University, Niigata 951-8514, Japan; 12 Division of Oral Biochemistry, Faculty of Dentistry, Graduate School of Medical and Dental Sciences, Niigata University, Niigata 951-8514, Japan; 13 Division of Microbiology and Infectious Diseases, Graduate School of Medical and Dental Sciences, Niigata University, Niigata 951-8514, Japan

**Keywords:** dental pulp cells, feeder cells, genetic engineering, human deciduous teeth, stem cells

## Abstract

Tissue-specific stem cells exist in tissues and organs, such as skin and bone marrow. However, their pluripotency is limited compared to embryonic stem cells. Culturing primary cells on plastic tissue culture dishes can result in the loss of multipotency, because of the inability of tissue-specific stem cells to survive in feeder-less dishes. Recent findings suggest that culturing primary cells in medium containing feeder cells, particularly genetically modified feeder cells expressing growth factors, may be beneficial for their survival and proliferation. Therefore, the aim of this study was to elucidate the role of genetically modified human feeder cells expressing growth factors in maintaining the integrity of primary cultured human deciduous dental pulp cells. Feeder cells expressing leukemia inhibitory factor, bone morphogenetic protein 4, and basic fibroblast growth factor were successfully engineered, as evidenced by PCR. Co-culturing with mitomycin-C-treated feeder cells enhanced the proliferation of newly isolated human deciduous dental pulp cells, promoted their differentiation into adipocytes and neurons, and maintained their stemness properties. Our findings suggest that genetically modified human feeder cells may be used to maintain the integrity of primary cultured human deciduous dental pulp cells.

## 1. Introduction

Stem cells are largely classified into two groups: embryonic stem cells/induced pluripotent stem cells (ESCs/iPSCs) and tissue-specific stem cells (TSCs) (also known as somatic stem cells or progenitor cells) [1,2,3,4,5]. The former possess self-renewal and pluripotency properties, and are derived from early embryos through continuous cultivation of the inner cell mass of blastocysts (ESCs) or from differentiated cells (i.e., fibroblasts) after transfection with reprogramming factors (Yamanaka factors) (iPSCs) [2]. The latter exist in tissues and organs, such as skin, brain, and bone marrow. TSCs possess self-renewal ability, but with limited pluripotency compared to ESCs/iPSCs. Importantly, ESCs/iPSCs (but not TSCs) can form solid tumors (teratomas) when transplanted under the skin or renal capsule of immunocompromised mice [6,7,8,9]. The role of stem cells in regenerative medicine has received considerable attention, with promising results.

Human deciduous teeth are discarded by children aged 6–12 years old, after which they are replaced by adult teeth. Stem cells can be isolated from human deciduous teeth, and dental pulp cells derived from human deciduous teeth (hereafter referred to as human deciduous dental pulp cells [HDDPCs]) may be a useful resource for tooth regeneration [10,11]. Therefore, a comprehensive understanding of the properties of HDDPCs is required. However, most primary cultured HDDPCs are maintained in feeder-less plastic tissue culture dishes, often leading to loss of pluripotency, probably due to the inability of TSCs to survive on feeder-less plastic tissue culture dishes for prolonged periods [12,13].

In 1981, mouse ESCs were first derived from teratocarcinoma stem cells cultured in medium containing mitotically inactivated mouse embryonic fibroblast (MEF) feeder cells supplemented with fetal bovine serum (FBS) [14,15]. Feeder cells are known to produce growth factors, adhesion molecules, and extracellular matrix components for cell attachment [16]. MEFs are primary cells derived from mid-gestational fetuses that can be maintained for only a few passages before senescence, necessitating the use of mitomycin-C (MMC; a reagent used to inhibit the proliferation of dividing cells) [17]. STO cells (an immortalized cell line derived from mouse SIM embryonic fibroblasts [18]) are useful for establishing and maintaining mouse [19,20] and human ESCs [2]. STO cells are also useful for eliminating bacterial contamination during HDDPC isolation and propagation [21]. In contrast to MEFs, STO cells can continue to proliferate in vitro; however, their proliferation can be inhibited by MMC or gamma irradiation [17]. Moreover, feeder cells can be engineered to express and secrete growth factors, such as leukemia inhibitory factor (LIF), bone morphogenetic protein-4 (BMP4), and basic fibroblast growth factor (bFGF; also known as FGF-2), which are important for establishing and maintaining mouse and human ESCs [22,23,24,25,26,27]. These findings indicate that the addition of recombinant growth factors to the medium is unnecessary when ESCs are co-cultured with genetically engineered feeder cells capable of producing growth factors. Furthermore, feeder cells carrying drug resistance genes are useful for selecting genetically engineered cells [20,22].

However, little is known about how primary cultured cells, such as HDDPCs, can be maintained in vitro. Therefore, the aim of this study was to elucidate the role of genetically modified human feeder cells in maintaining the integrity of primary cultured HDDPCs. We hypothesized that co-culturing with feeder cells is important for maintaining the integrity of newly isolated HDDPCs. Considering the future use of HDDPC-derived stem cells in preclinical studies, human-derived feeder cells were used in this study to avoid xeno-contamination. Because human-derived feeder cells can be genetically engineered to express and secrete growth factors, we generated genetically modified (GM) HDDPCs through co-transfection with genes encoding LIF, BMP4, and bFGF, using a *piggyBac* (*PB*) transposon-based gene delivery system [28,29]. The role of GM HDDPCs in the growth, differentiation, and stemness properties of newly isolated HDDPCs was examined.

## 2. Materials and Methods

### 2.1. Cells

HDDPCs were isolated from human deciduous recovered from children aged 6–12 years old by digestion in a solution of 3 mg/mL of collagenase type I (#17100-017; Invitrogen, Carlsbad, CA, USA) and 4 mg/mL of dispase (#410810077; Roche Applied Science, Basel, Switzerland) for 30–60 min at 37 °C. The isolated HDDPCs were maintained in 60-mm gelatin-coated dishes (#4010-020; Iwaki Glass Co., Ltd., Tokyo, Japan) containing minimum essential medium α (MEMα) supplemented with L-glutamine and phenol red (#135-15175; Wako Pure Chemical Industries, Ltd., Osaka, Japan), 20% heat-inactivated FBS (#SFMB30-2239; Equitech Bio Inc., Kerrville, TX, USA), 50 U/mL of penicillin, and 50 mg/mL of streptomycin (#15140-122; Invitrogen) (hereafter referred to as MEMα/20% FBS) at 37 °C under 5% CO_2_ for >7 d. The medium was changed every 3 days. Primary cultured cells were obtained after approximately 10 passages and frozen using CellBanker cell freezing medium (#CB021; Takara Bio Inc., Shiga, Japan). The isolated HDDPCs were used in co-cultivation assays with MMC-treated GM HDDPCs. HDDPCs [29] that had been passaged for 20 generations, were used as GM feeder cells. These cells were maintained under the same conditions as the newly isolated HDDPCs.

All HDDPC experiments were performed according to the guidelines and protocols approved by the Ethical Committee for the Use and Experimentation of Graduate School of Dental Science, Asahi University (No. 34007; dated on 16 March 2022).

### 2.2. Construction of PB Transposon Vectors

**Figure 1:**
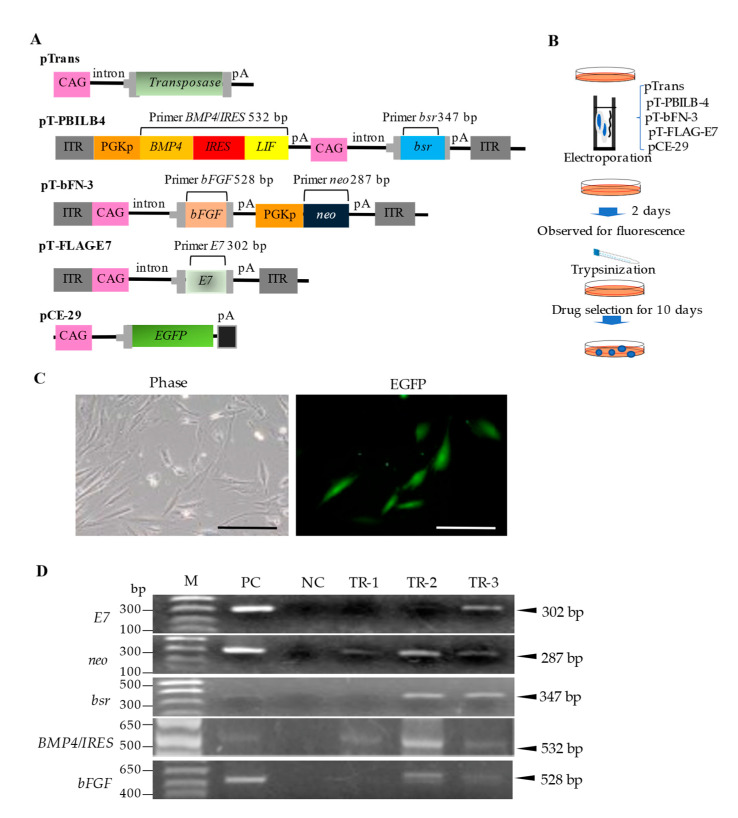
Isolation of GM HDDPCs via transfection using a *PB* transposon-based gene delivery system. (**A**) Plasmid construction. pTrans is a vector for the expression of *PB* transposase under the control of the CAG promoter. pT-bFN-3 is a *PB*-based vector that carries a *bFGF* expression unit under the control of the CAG promoter and a *neo* expression unit under the control of the mouse Pgk promoter. pT-PBILB-4 is a *PB*-based vector that carries a *BMP4*/*LIF* expression unit under the control of the mouse Pgk promoter and a *bsr* expression unit under the control of the CAG promoter. pT-FLAG-E7 is a *PB*-based vector that carries a bovine papilloma virus-derived *E7* expression unit under the control of the CAG promoter. pCE-29 is a plasmid vector for the expression of *EGFP* cDNA under the control of the CAG promoter. (**B**) Flowchart of the isolation procedure. (**C**) Fluorescence 2 d after transfection. Phase, photograph taken under light; EGFP, photograph taken under UV light. Scale bars, 50 μm. (**D**) PCR analysis of transfected clones (TR-1–3). Abbreviations: *bFGF*: basic fibroblast growth factor; *BMP4*: bone morphogenetic protein-4; *bsr*: blasticidin S-resistance gene; CAG: chicken β-actin promoter; *EGFP*: enhanced green fluorescent protein; GM: genetically modified; HDDPC: human deciduous dental pulp cells; *IRES*: internal ribosomal entry site; ITR: inverted terminal repeat; *LIF*: leukemia inhibitory factor; M: molecular size markers; NC: negative control (genomic DNA from non-transfected HDDPCs); pA: poly (A) addition site; *PB*: *piggyBac*; PC: positive control (5 ng of each plasmid); Pgk: phosphoglycerate kinase; Pgkp: Pgk promoter.

*PB*-based expression vectors (Figure 1A) were constructed using a standard cloning procedure. pTrans [30] is a vector for the expression of *PB* transposase under the control of the chicken β-actin (CAG) promoter [31]. pT-bFN-3 is a *PB*-based vector that carries a *bFGF* expression unit under the control of the CAG promotor and a neomycin resistance gene (*neo*) expression unit under the control of the mouse phosphoglycerate kinase (Pgk) promoter. pT-PBILB-4 is a *PB*-based vector that carries a *BMP4*/*LIF* expression unit under the control of the mouse Pgk promoter and a blasticidin S-resistance gene (*bsr*) expression unit under the control of the CAG promoter. *BMP4*/*LIF* expression is mediated by the internal ribosome entry site, a multicistronic element enabling CAP-independent protein translation [32]. pT-FLAG-E7 is a *PB*-based vector that carries a bovine papilloma virus-derived *E7* expression unit under the control of the CAG promoter. *E7* is an immortalization gene responsible for continuous cell proliferation. pCE-29 [33] is a plasmid vector for the expression of enhanced green fluorescent protein (*EGFP*) cDNA under the control of the CAG promoter. All plasmids were grown in *E. coli* DH5α and purified using a Macherey-Nagel plasmid purification kit as previously described [34].

### 2.3. Generation of GM HDDPCs via Transfection with PB-Based Vectors

HDDPCs [26] (5 × 10^5^ cells) were electroporated in 100 µL of R solution (Invitrogen) containing pTrans, pT-bFN-3, pT-PBILB-4, pT-FLAG-E7, and pCE-29 (1 μg each) using the Neon Transfection System (#MPK10096; Invitrogen), as shown in Figure 1B. The cells were examined for EGFP-derived fluorescence 2 d after transfection. The cells were then trypsinized and reseeded in 60-mm gelatin-coated dishes containing MEMα/20% FBS supplemented with 800 μg/mL of G418 (#631307; TaKaRa Bio Inc.) and 400 μg/mL of *bsr* (#3513-03-9; Merck, Darmstadt, Germany). After 7−10 d of selection, the emerging colonies were picked using a 3-mm paper disc (#3030-917; Whatmann, Buckinghamshire, UK) dipped in 0.25% trypsin in calcium- and magnesium-free Dulbecco’s modified phosphate-buffered saline (DPBS), as previously described [29]. The cell-containing paper discs were transferred to a gelatin-coated 48-well plate (#3830-048; Iwaki Glass Co., Ltd.) containing 200 μL of drug-free MEMα/20% FBS and cultured for 7–10 d at 37 °C under 5% CO_2_. The cells were further propagated in a stepwise manner and frozen for molecular immunocytochemical analysis.

### 2.4. Preparation of MMC-Treated Feeder Cells

GM HDDPCs (10^5^ cells) (Section 2.3) were incubated in a 60-mm gelatin-coated dish containing 4 mL of MEMα/20% FBS supplemented with 4 μg/mL of MMC (#M4287; Sigma-Aldrich, St. Louis, MO, USA), for 4 h at 37 °C under 5% CO_2_, to inhibit cell proliferation. After treatment, cells were washed three times with DPBS, trypsinized, aliquoted (10^4^ dissociated cells) into cryotubes (#430488; Corning, Glendale, AZ, USA) containing 400 μL of CellBanker (#CB021; TaKaRa Bio Inc.), and deep-frozen.

### 2.5. PCR Analysis

Genomic DNA was extracted by adding 300 μL of lysis buffer [0.125 μg/mL of proteinase K, 0.125 μg/mL of Pronase E, 0.32 M sucrose, 10 mM Tris-HCl (pH 7.5), 5 mM MgCl_2_, and 1% (*v*/*v*) Triton X-100] to transfected HDDPCs (~10^5^ cells) in a 1.5-mL tube, followed by incubation for 2–3 d at 37 °C and phenol/chloroform extraction. The supernatant was isopropanol precipitated. The precipitated DNA was then dissolved in 20 μL of sterile water and stored at 4 °C. PCR was performed in a total reaction volume of 10 μL containing 10 mM Tris-HCl (pH 8.3), 50 mM KCl, 1.5 mM MgCl_2_, 0.25 mM of each dNTP, 1 mM of each primer (forward and reverse) (Table 1), 2 μL of genomic DNA (~5 ng), and 0.5 U of rTaq polymerase (#R001; Takara Shuzo Co., Ltd., Tokyo, Japan). The PCR conditions were as follows: 40 cycles at 96 °C for 10 s, 56 °C for 1 min, and 72 °C for 2 min. *E7*-S and *E7*-RV primers were used to detect pT-FLAG-E7, which yielded a 302-bp product from the upper region of *E7*. Similarly, *neo*-3S and *neo*-3RV primers were used to detect the *neo* expression unit in pT-bFN-3, which yielded a 287-bp product from *neo*. Additionally, *bsr*-S and *bsr*-RV primers were used to detect the *bsr* expression unit in pT-PBILB-4, which yielded a 347-bp product from *bsr*. *BMP4*/*LIF*-S and *BMP4*/*LIF*-RV primers were used to detect the fragment containing the *BMP4/LIF* expression unit in pT-PBILB-4, which yielded a 532-bp product from *BMP4/LIF*. *bFGF*-S and *bFGF*-RV primers were used to detect the *bFGF* expression unit in pT-bFN-3, which yielded a 528-bp product from *bFGF*. Genomic DNA (0.5 μg) from untransfected HDDPCs was used as a negative control; 5 ng of each plasmid listed in Figure 1A were used as positive controls. The PCR products (5 µL) were separated on a 2% agarose gel and visualized with ethidium bromide.

### 2.6. Immunocytochemical Staining

Immunocytochemical staining was performed to detect the transgenic products. Briefly, GM HDDPCs were seeded in a gelatin-coated 24-well plate (#3820-024; Iwaki Glass Co., Ltd.) containing MEMα/20% FBS. Cells at 60–70% confluence were fixed in 4% paraformaldehyde (PFA) in DPBS for 5 min at 24 °C, washed three times with DPBS, and permeabilized with 0.1% Triton X-100 (#T8787; Sigma-Aldrich) in DPBS for 3 min at 24 °C. Cells were washed three times with DPBS containing 1% normal goat serum (NGS) (Invitrogen) (hereafter referred to as PBS/NGS) and blocked by incubation with 20% AquaBlock (#PP82; East Coast Biologics, Inc., North Berwick, USA) for 30 min at room temperature. Cells were washed three times with PBS/NGS and stained with primary antibodies against BMP4 (clone H-134, 1:200) (#sc-9081; Santa Cruz Biotechnology, Dallas, TX, USA), LIF (1:200) (#SAB2701974; Sigma-Aldrich), or bFGF (1:200) (#N5413; Sigma-Aldrich) overnight at 4 °C. After washing 3 times with PBS/NGS, the cells were incubated with fluorescein isothiocyanate-conjugated goat anti-mouse immunoglobulin G γ-chain secondary antibody (1:200) (#AP124JA4; Millipore-Chemicon, Darmstadt, Germany) for approximately 2 h at 4 °C. After washing with PBS/NGS 3 times, nuclear staining was performed with 4′,6-diamidino-2-phenylindole (#H-1200; Vector Laboratories, Burlingame, CA, USA) for 10 min at room temperature. Fluorescence was examined using an Olympus BX60 fluorescence microscope (#BX60; Olympus, Tokyo, Japan).

### 2.7. Cell Growth Assay

To determine the effect of MMC-treated GM HDDPCs on the proliferation of freshly isolated HDDPCs, frozen stocks of MMC-treated GM HDDPCs were thawed 4 d prior to the cell growth assay. For the experimental group, MMC-treated GM HDDPCs (10^4^ cells) and freshly isolated HDDPCs (4 × 10^4^ cells) were mixed at a ratio of 1:5 in a volume of 180 μL. A 10 μL aliquot of cell suspension was plated on a gelatin-coated 24-well plate containing 1 mL of drug-free MEMα/20% FBS as the first passage. After culturing for 6–10 d, cells were harvested via trypsinization and counted using a disposable hemocytometer (#521-10; Funakoshi, Tokyo, Japan). The final cell number was calculated by subtracting the number of MMC-treated GM HDDPCs (10^4^ cells) initially plated from the total cell number. Two wells per line were examined and the average cell number was plotted. Cells were mixed with MMC-treated GM HDDPCs (10^4^ cells), pelleted, and resuspended in 180 μL of drug-free MEMα/20% FBS. A 10 μL aliquot of cell suspension was plated on a 24-well plate as the second passage. This was repeated for up to 10 passages. For the control group, freshly isolated HDDPCs (5 × 10^4^ cells) alone were prepared in a volume of 180 μL. A 10 μL aliquot of cell suspension was plated on a gelatin-coated 24-well plate containing 1 mL of drug-free MEMα/20% FBS as the first passage. After culturing for 6–10 d, cells were harvested via trypsinization and counted using a hemocytometer (#521-10; Funakoshi Co., Ltd., Tokyo, Japan). Two wells per line were examined and the average cell number was plotted. Cells were pelleted and resuspended in 180 μL of drug-free MEMα/20% FBS. A 10 μL aliquot of cell suspension was plated on a 24-well plate as the second passage. This was repeated for up to 10 passages.

### 2.8. In vitro Differentiation Assay

To determine the effect of MMC-treated GM HDDPCs on the differentiation of freshly prepared HDDPCs, frozen stocks of MMC-treated GM HDDPCs were thawed 4 d prior to the assay. For the experimental group, MMC-treated GM HDDPCs (1 × 10^4^ cells) and freshly isolated HDDPCs (5 × 10^4^ cells) were mixed at a ratio of 1:5 in a volume of 180 μL. A 100 μL aliquot of cell suspension was plated on a gelatin-coated 24-well plate containing 1 mL of drug-free MEMα/20% FBS. For the control group, freshly isolated HDDPCs (6 × 10^4^ cells) alone were prepared in a volume of 180 μL. A 100 μL aliquot of cell suspension was plated on a gelatin-coated 24-well plate containing 1 mL of drug-free MEMα/20% FBS. When the cells reached 80–90% confluence, the medium was changed to a differentiation-inducing medium, as described below.

To induce osteogenic differentiation, cells were cultured in osteogenic differentiation medium (#KBDSTC103; DS Pharma, Osaka, Japan) for ~5 d at 37 °C under 5% CO_2_. Cells were fixed in 4% PFA for 5 min at 24 °C and washed three times with DPBS, followed by Alizarin Red S staining (#ARD-A1; PG Research, Tokyo, Japan) for 30 min at room temperature.

To induce neurogenic differentiation, cells were cultured in mesenchymal stem cell neurogenic differentiation medium (#C-28015; PromoCell, Heidelberg, Germany) for 7 day at 37 °C under 5% CO_2_. After fixation in 4% PFA for 5 min at room temperature, the cells were incubated with 0.1% Cresyl violet solution (#038-0482; Wako Pure Chemical Industries Ltd.) for 30 min at room temperature to stain the cytoplasm of neurons with Nissl.

### 2.9. Alkaline Phosphatase (ALP) Assay

To determine the effect of MMC-treated GM HDDPCs on ALP expression by freshly prepared HDDPCs, frozen stocks of MMC-treated GM HDDPCs were thawed 4 d prior to the assay. For the experimental group, MMC-treated GM HDDPCs (1 × 10^3^ cells) were seeded in a gelatin-coated 24-well plate containing 1 mL of drug-free MEMα/20% FBS, followed by the addition of freshly isolated HDDPCs (5 × 10^3^ cells) and cultured for 7 d. After trypsinization, the cell suspension (~5 × 10^3^ cells) was reseeded in a gelatin-coated 24-well plate containing MMC-treated GM HDDPCs (1 × 10^3^ cells) and cultured for 7 d. This procedure was repeated for 20 passages. For the control group, freshly isolated HDDPCs (~5 × 10^3^) alone were seeded in a gelatin-coated 24-well plate and cultured for 7 d. After trypsinization, the cell suspension (~5 × 10^3^ cells) was reseeded in a gelatin-coated 24-well plate. This was repeated for at least 20 passages. Cells at 80–90% confluency were fixed in 4% PFA for 5 min at 24 °C, followed by cytochemical staining for ALP activity using the Leukocyte Alkaline Phosphatase Kit (#ALP-TK1; Sigma-Aldrich, St. Louis, MO, USA). ALP activity was visualized by the appearance of red-brown products upon ALP-mediated conversion of α-naphthol coupled with a diazonium salt.

## 3. Results

### 3.1. Preparation of GM HDDPCs

HDDPCs were transfected with *PB*-based transposon vectors (Figure 1A), which are effective for acquiring stable mammalian transfectants [28], to generate stable transfectants expressing several growth factors and an immortalization gene. HDDPCs were electroporated with a *PB* transposase expression vector, pTrans, three *PB* transposons (pT-bFN-3, pT-PBILB-4, and pT-FLAG-E7), and an *EGFP* expression vector, pCE-29 (used to monitor transfection efficiency), using the Neon Transfection System (Figure 1B). Approximately 40% of the cells (1/2 tested) exhibited *EGFP*-derived fluorescence after 2 d of transfection (Figure 1C). To acquire stable transfectants, transfected cells were treated with G418 and *bsr* for 7–10 d. The emerging colonies were picked using a trypsin-dipped paper disc, as described by Nakayama et al. [35], transferred to a 48-well plate containing drug-free MEMα/20% FBS, and propagated for 7–10 d. Three clones were successfully obtained (TR-1–3).

**Figure 2:**
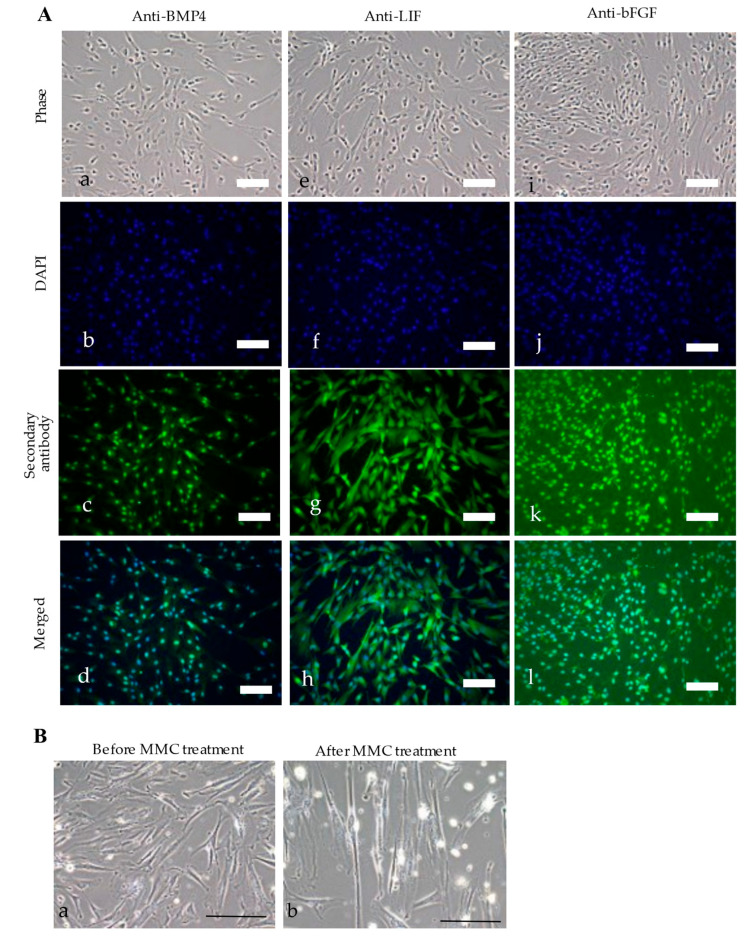
Characterization of TR-2 feeder cells. (**A**) Immunocytochemical staining of TR-2 feeder cells. Cells were immunostained with (a–c), and (d) anti-BMP, (e–h) anti-LIF, or (i), (j), (k), and (l) anti-bFGF antibodies. Scale bars = 50 μm. (**B**) Morphological changes in TR-2 feeder cells (a) before and (b) 35 d after MMC treatment. Scale bars = 100 μm. bFGF: basic fibroblast growth factor; BMP4: bone morphogenetic protein-4; DAPI: 4′,6-diamidino-2-phenylindole; LIF: leukemia inhibitory factor; MMC: mitomycin-C.

PCR was performed to confirm the presence of the introduced genes in the established clones. TR-1 contained *neo* and *BMP4/LIF*, whereas both TR-2 and TR-3 contained *E7, neo, bsr, BMP4/LIF*, and *bFGF* (Figure 1D). TR-2 had a higher proliferation rate in vitro than TR-3; therefore, TR-2 was selected for subsequent experiments. Immunocytochemical staining using antibodies against BMP4, LIF, and bFGF confirmed the expression of these proteins in TR-2 (Figure 2A).

TR-2 was incubated in MEMα/20% FBS containing MMC for 4 h at 37 °C to determine the effect of MMC on TR-2 morphology. There was no change in cell behavior (e.g., detachment from the dish surface); however, MMC-treated TR-2 (hereafter referred to as “TR-2 feeder cells”) exhibited a slightly enlarged morphology (Figure 2B).

### 3.2. HDDPC-Derived Feeder Cells Enhanced the Proliferation of HDDPCs

**Figure 3:**
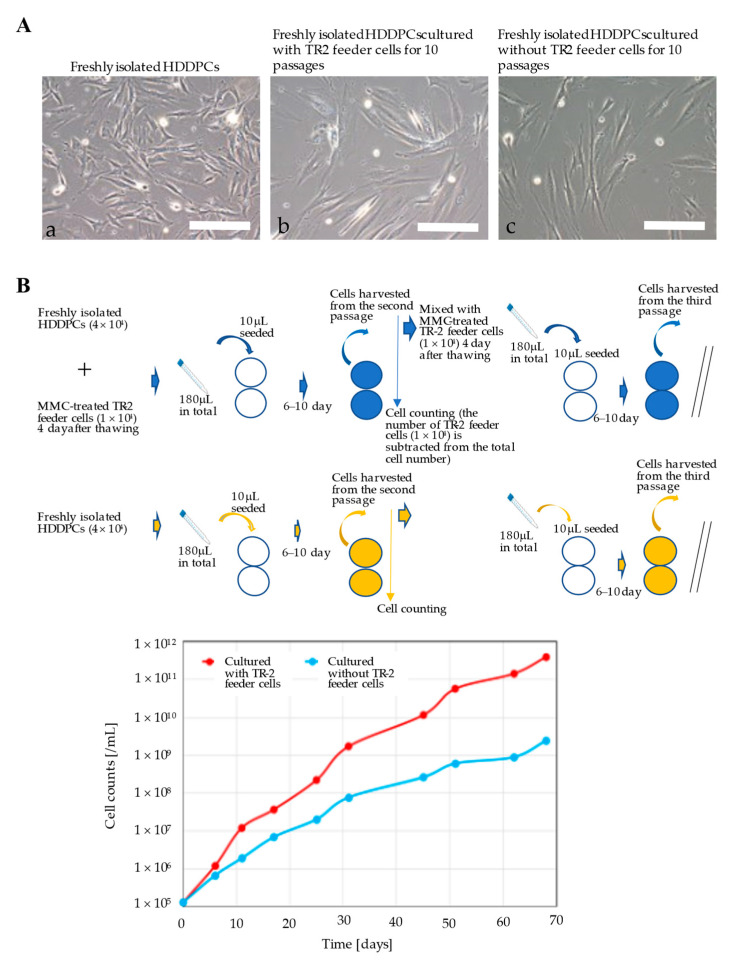
Characterization of freshly isolated primary HDDPCs cultured with or without TR-2 feeder cells (I). (**A**) Morphology of primary HDDPCs (a) before transfection, (b) cultured with TR-2 feeder cells for 10 passages, and (c) cultured without TR-2 feeder cells for 10 passages. Scale bars = 100 μm. (**B**) Proliferation of primary HDDPCs with or without MMC-treated TR-2 feeder cells. The assay is shown schematically in the upper column. In the lower column, the proliferation rate after 10 passages is shown. Primary HDDPCs cultured with TR-2 feeder cells had a higher proliferation rate than those cultured without TR-2 feeder cells. HDDPC: human deciduous dental pulp cells; MMC: mitomycin-C.

Primary HDDPCs were cultured with or without TR-2 feeder cells, and morphological changes were evaluated after 10 passages. In the presence or absence of TR-2 feeder cells, the morphology of HDDPCs remained fibroblastic after 4 d of culture (nine passages) (Figure 3A). However, HDDPCs cultured with TR-2 feeder cells had a higher proliferation rate than those cultured without TR-2 feeder cells (Figure 3B), indicating that TR-2 feeder cells enhanced the proliferation of HDDPCs.

### 3.3. HDDPC-Derived Feeder Cells Maintained the Pluripotency of HDDPCs

**Figure 4:**
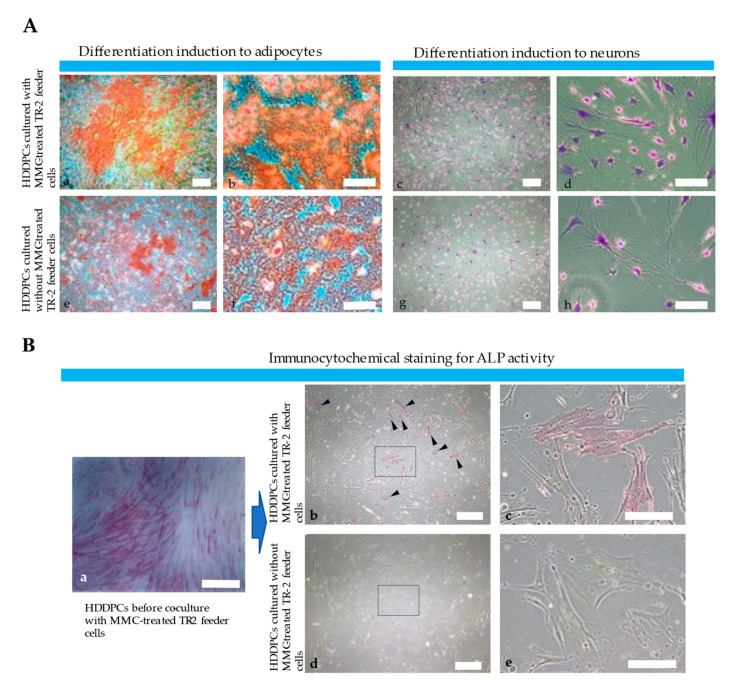
Characterization of freshly isolated primary HDDPCs cultured with or without TR-2 feeder cells (II). (**A**) Osteogenic and neurogenic differentiation of primary HDDPCs cultured in the (a–c), and (d) presence or (e–g), and (h) absence of TR-2 feeder cells. (a,b,e,f) Alizarin red staining 5 d after incubation in osteogenic medium. (c,d,g,h) Nissl staining 7 d after incubation in neurogenic medium. HDDPCs cultured with TR-2 feeder cells exhibited better differentiation than those cultured without TR-2 feeder cells. Scale bars = 100 μm [(a,c,e,g)] and 50 μm [(b,d,f,h)]. (**B**) Expression of ALP (a stem cell marker). Cytochemical ALP staining of HDDPCs (a) before culturing with TR-2 feeder cells, (b,c) cultured with TR-2 feeder cells for 20 passages, and (d,e) cultured without TR-2 feeder cells for 20 passages. The former maintained ALP activity [arrowheads (b)]. The boxes in (b,d) are enlarged and shown as (c,e), respectively. Scale bars = 100 μm [(a,b,d)] and 50 μm [(c,e)]. ALP: alkaline phosphatase; HDDPC: human deciduous dental pulp cells; MMC: mitomycin-C.

Primary HDDPCs were cultured with or without TR-2 feeder cells for 10 passages, and then subjected to osteogenic or neurogenic differentiation induction to determine whether TR-2 feeder cells supported the pluripotency of HDDPCs. HDDPCs cultured with TR-2 feeder cells exhibited higher osteogenic differentiation (as determined by the appearance of heavily calcified deposits) after 5 d of culture than those cultured without TR-2 feeder cells (Figure 4A). Similarly, HDDPCs cultured with TR-2 feeder cells exhibited higher neurogenic differentiation after 7 d of culture than those cultured without TR-2 feeder cells (as evidenced by the greater number of elongated axons and dendrites with Nissl bodies around the nucleus compared to those cultured without TR-2 feeder cells) (Figure 4A).

### 3.4. HDDPC-Derived Feeder Cells Maintained the Stemness of HDDPCs

Previously, we demonstrated that HDDPCs with high ALP, OCT3/4, and SOX2 expression are easily reprogrammed into iPSCs, with pluripotent capabilities [29], indicating that HDDPCs with high ALP activity have stemness properties. Based on these findings, we performed immunocytochemical staining of ALP in HDDPCs cultured with or without TR-2 feeder cells for 20 passages. HDDPCs cultured with TR-2 feeder cells exhibited mosaic ALP staining (Figure 4B), like parental HDDPCs, indicating a mixture of stem and non-stem cells. Conversely, HDDPCs cultured without TR-2 feeder cells exhibited negative ALP staining (Figure 4B). Our findings suggest that HDDPCs cultured with TR-2 feeder cells possess stemness properties and can be easily reprogrammed into iPSCs.

## 4. Discussion

Since the first establishment of mouse ESCs using mitotically inactivated feeder cells in 1981 [36,37], mitosis-incompetent murine or human embryonic fibroblast cells have been used to generate and maintain human and rat ESCs/iPSCs and mouse iPSCs [2,3,38,39,40,41,42]. Additionally, feeder cells are effective in maintaining various types of juvenile cells, such as hematopoietic progenitor cells [11,12,13,14], limbal epithelial progenitor cells [43,44,45,46], spermatogonial progenitor cells from adult mammalian testis [7,47], and stem cells from human corneal or oral epithelial cells [5,6]. Although MEFs are commonly used as feeder cells, there have been no reports of the creation of feeder cells that are more suitable for stem cell maintenance than MEFs. In this study, we produced feeder cells derived from HDDPCs that have enough ability to maintain undifferentiated cells.

Continuous culture in feeder-less plastic tissue culture dishes can result in the gradual loss of pluripotency of TSCs [15,16] and ESCs/iPSCs [48]. Since the first report that the cytokine LIF can maintain the self-renewal and pluripotency of mouse ESCs in the absence of feeder cells [19,49], the beneficial effects of adding specific growth factors, such as BMP4 and bFGF, into the culture medium of ESCs/iPSCs have been reported [50,51]. Researchers have generated genetically engineered cells overexpressing growth factors to achieve cost-effective ESCs/iPSCs culture systems [52,53]. Horie et al. [54] demonstrated that GM fibroblasts (STO cells) expressing E-cadherin (an adhesion molecule involved in cell-to-cell interactions) [55] maintained the pluripotency of mouse ESCs, indicating the importance of scaffold formation between feeder cells and ESCs. In this study, GM HDDPCs (TR-2 feeder cells) simultaneously expressing three growth factors (bFGF, LIF, and BMP4) and an immortalization gene were generated to support the proliferation and differentiation of primary HDDPCs. Furthermore, 10% FBS has been frequently employed for maintaining differentiated cells, but when cultivating cells enriched with stem cells, 20% FBS has been preferred in order to achieve stable growth rates [56]. Notably, contamination with oral bacteria was common during the primary culture of HDDPCs. To prevent contamination, antibiotics were used consistently in our experiments, and they did not appear to affect the pluripotency of HDDPCs.

In this study, a *PB*-based gene delivery system was used to construct TR-2 feeder cells, enabling the isolation of stable transfectants from various cell types [57]. *PB*-based gene delivery is simple because researchers can use only two types of nucleic acids: a *PB* transposase (Trans) expression vector and a transposon vector carrying a gene of interest flanked by two inverted terminal repeats. When the nucleic acids are placed inside a cell, *PB* Trans binds to the inverted terminal repeats to allow the gene of interest to be individually integrated into host chromosomal sites containing the TTAA sequence, which is duplicated on the two flanks of the integrated fragment [58,59]. Dental pulp cells have been used as a material for gene transfection, and bone differentiation can be promoted by introducing *OCT3/4* and other genes [60]. In this study, four constructs (three transposons and one non-transposon plasmid) were introduced into HDDPCs in vitro and three clones (TR-1–3) were obtained. Among the clones, TR-2 was found to contain all four genes of interest (*BMP4*, *LIF*, *bFGF*, and *E7*) (Figure 1B) in its genome, and successfully expressed BMP4, LIF, and bFGF via immunocytochemical assay (Figure 2A). These results show that the *PB*-based gene delivery system was effective in generating GM HDDPCs with multiple genes.

Notably, HDDPCs obtained after 1–4 passages often showed unstable growth rates, possibly due to the various types of cells included with the HDDPCs. After culturing in the conditions used in this study, HDDPCs retained stem-like properties even after 10 passages [61], and exhibited stable growth rates [62]. The growth assay shows that, after 10 passages, HDDPCs co-cultured with TR-2 feeder cells had a higher proliferation rate than those cultured without TR-2 feeder cells (Figure 3B). HDDPCs cultured with TR-2 feeder cells had better differentiation potential than those cultured without TR-2 feeder cells (Figure 4A), indicating that GM human feeder cells are important for maintaining the integrity of primary cultured HDDPCs. Continuous culture of primary HDDPCs in feeder-less medium resulted in a loss of ALP activity (Figure 4B), indicating a loss of stem cells (initially included in the isolation of HDDPCs). A previous study showed that primary HDDPCs comprise various cells, including stem and non-stem cells [63]. As ALP and OCT-3/4 are markers of ESCs/iPSCs [34,59,63], ALP(−)/OCT-3/4(−) cells may be considered non-stem cells, whereas ALP(−)/OCT-3/4(+), ALP(+)/OCT-3/4(−), and ALP(+)/OCT-3/4(+) cells may be considered stem cells [63]. This study indicates that the secretion of growth factors from TR-2 feeder cells may be beneficial for stem cell survival. Notably, expression of CD73, CD90, and STRO-1, all of which are recognized as markers for mesenchymal stem cells (MSCs), has been reported in HDDPCs [64]. Unfortunately, we did not test for expression of these proteins in the HDDPCs used in the present study. HDDPCs should be comprehensively checked for the expression of stem-cell-specific markers, including MSC-related markers, in future studies.

The use of feeder cells is labor intensive, as the feeder layer needs to be seeded a day before stem cell culture, and MMC treatment or gamma irradiation is required to inhibit cell proliferation. Therefore, an alternative feeder-free culture system for human ESCs/iPSCs has been developed using a medium containing 2i (GSK3 and MAPK inhibitor) and LIF [65], heparin [66], DNA aptamer capable of binding to a bFGF receptor [67], or human plasma protein-based hydrogel [68]. Commercially available media (StemFlex [#A3349401; Thermo Fisher Scientific], mTeSR™1 [#ST-85850; Veritas Japan Co., Ltd., Yokohama, Japan], and StemFit Basic04 Complete Type (#ASB04CT; Ajinomoto, Tokyo, Japan) can also be used to culture human ESCs/iPSCs. Human ESCs/iPSCs were established or maintained on tissue culture surface coated with extracellular matrix proteins, such as fibronectin, vitronectin, and laminin [65,69], or on commercially available Matrigel (#354234; Corning, NY, USA), which is liquid at low temperatures and a gel at 37 °C [65,70]. Porous membranes [71], synthetic substrates [72], and DAS nanocrystalline graphene [73] have also been used in feeder-free culture systems for human ESCs/iPSCs. To our knowledge, there have been few reports on the successful cultivation of human dental pulp-derived stem cells in the absence of feeder cells. Although some reagents have been developed to support these juvenile cells, their mechanisms of action are unclear, and they may be expensive. Therefore, GM feeder cells may serve as an effective and cheap alternative for maintaining stem cells.

## 5. Conclusions

Our findings show that co-cultivation of primary HDDPCs with MMC-treated TR-2 feeder cells can maintain the integrity of primary HDDPCs, including proliferation rate, differentiation ability, and expression of stemness markers. As TR-2 feeder cells express growth factors that are potentially beneficial for maintaining juvenile cells, such as stem cells, progenitor cells, and ESCs/iPSCs, they may be useful for maintaining other types of juvenile cells that are difficult to maintain in the absence of feeder cells or require specific growth medium.

## Figures and Tables

**Table 1:** PCR primers.

Primer (Orientation)	Target Gene	Sequence (5′–3′)
*E7*-S (sense)	*E7*	CTC CTG GGC AAC GTG CTG GT
*E7*-RV (reverse)	*E7*	TGG CTT CAC ACT TAC AAC ACA
*neo*-3S (sense)	*neo*	TTT CTG GAT TTG CAG GTG AAC
*neo*-3RV (reverse)	*neo*	GTG ATG TCC AGC TTG GTG TCC
*bsr*-S (sense)	*bsr*	TCT ACG AGC GGC TCG GCT TCA
*bsr*-RV (reverse)	*bsr*	TCA GGC ACC GGG CTT GCG GGT
*BMP4*/*IRES*-S (sense)	*IRES*	TGT ACC TGG ATG AGT ATG AT
*BMP4/IRES*-RV (reverse)	*IRES*	CCT CAC ATT GCC AAA AGA CG
*bFGF*-S (sense)	*bFGF*	TCT ACG AGC GGC TCG GCT TCA
*bFGF*-RV (reverse)	*bFGF*	TCA GGC ACC GGG CTT GCG GGT

*E7*: *E7* gene from human papillomavirus type 18; *neo*: neomycin resistance gene; *bsr*: blasticidin S-resistance gene; *IRES*: internal ribosomal entry site; *bFGF:* basic fibroblast growth factor.

## Data Availability

Not applicable.
